# Loud Call Production in Male Vervet Monkeys (*Chlorocebus pygerythrus*) Varies with Season and Signaller Rank

**DOI:** 10.1007/s10764-024-00475-x

**Published:** 2025-01-23

**Authors:** Lukas Schad, Pooja Dongre, Erica van de Waal, Julia Fischer

**Affiliations:** 1 Department for Primate Cognition, Georg-August-Universität Göttingen, Göttingen, Germany; 2 Cognitive Ethology Laboratory, German Primate Center - Leibniz Institute for Primate Research, Göttingen, Germany; 3 Leibniz ScienceCampus Primate Cognition, Göttingen, Germany; 4 Department of Ecology and Evolution, University of Lausanne, Lausanne, Switzerland; 5 Centre for Functional Biodiversity, School of Life Sciences, University of KwaZulu-Natal, Pietermaritzburg, South Africa; 6 The Sense Innovation and Research Center, Lausanne and Sion, Switzerland; 7 Inkawu Vervet Project, Mawana Game Reserve, KwaZulu-Natal, South Africa

**Keywords:** Alarm calls, Loud calls, Sexual selection, Vocal communication, Quality handicap, Conventional signal

## Abstract

Adult males of many nonhuman primate species utter characteristic loud calls that are audible over long distances and serve various functions. In response to large terrestrial predators, male vervet monkeys (*Chlorocebus pygerythrus*) produce loud ‘barks’ that alert group members and are assumed to deter predators. It is less well known that male vervet monkeys also produce barks during aggressive interactions within and between groups. Here, we investigated whether barks potentially serve a dual function as alarm calls and quality signals. We used ad-libitum event sampling to record natural barking events from 45 adult males in six free-ranging groups over 24 months at Mawana Game Reserve, KwaZulu-Natal, South Africa. For each barking event in groups with multiple males, we aimed to record whether each adult male in the group barked or not. Using generalised linear mixed models, we investigated whether the number of barking events recorded per observation day and the probability that an individual male barked in each event were associated with male rank, the number of males in the group, the adult sex ratio, and the mating season. The highest-ranking males showed a very high probability of barking, and the number of daily barking events increased during the mating season. Males frequently barked in aggressive interactions, supporting the hypothesis that this signal plays a role in male–male competition. We conclude that, in addition to serving as alarm calls, barks may also advertise male competitive ability or motivation to compete.

**Supplementary Information:**

The online version contains supplementary material available at 10.1007/s10764-024-00475-x.

## Introduction

Many primate species produce ’loud calls’, conspicuous vocalisations that have high amplitudes and exhibit structural characteristics that reduce acoustic attenuation and facilitate sound propagation, rendering them audible over long distances (Delgado, 2006; Gautier & Gautier-Hion, 1977; Mitani & Stuht, 1998; Waser & Brown, 1986; Wich & Nunn, 2002). Researchers have proposed various functions for loud calls, including resource defence, mediating intra-group cohesion and inter-group spacing, or deterring predators and alerting group members (Briseño-Jaramillo *et al.*, 2017; Da Cunha & Byrne, 2006; Da Cunha & Jalles-Filho, 2007; Erb *et al.*, 2013, 2016; Fuller & Cords, 2017; Harris, 2006; Harris *et al.*, 2006; Isbell & Bidner, 2016; Zuberbühler *et al.*, 1997, 1999; Zuberbühler, 2002). Since in many species only adult males produce loud calls, they have also received attention as potential targets of sexual selection, implying that they may fulfil a function in mate attraction or male-male competition (Benítez *et al.*, 2016; Fischer *et al.*, 2004; Fuller & Cords, 2017, 2020; Harris *et al.*, 2006; Kitchen *et al.*, 2003; Neumann *et al.*, 2010; Snowdon, 2004; Zuberbühler, 2002).

Physiological constraints affecting signal structure or costs related to signal production can impose limitations on individual vocal structure and call rate, rendering loud calls potential honest signals advertising male condition and competitive ability (Maynard Smith & Harper, 2003; Vehrencamp, 2000). In several primate species, loud call structure and usage vary with signaller rank, suggesting a link between individual vocal behaviour and competitive ability (Benítez *et al.*, 2016; Fischer *et al.*, 2004; Kitchen *et al.*, 2003; Mitani & Nishida, 1993; Neumann *et al.*, 2010; Riede *et al.*, 2007). Although loud calls are audible to many receivers that may represent competitors, potential mates, or predators for the signaller, researchers have not explored potential dual functions in many species (Fuller & Cords, 2017, 2020; Zuberbühler, 2002; Zuberbühler *et al.*, 1997).

Vervet monkeys (*Chlorocebus pygerythrus*) produce structurally distinct vocalisations in response to their main predator categories (large mammalian carnivores, snakes, and eagles) that elicit anti-predator behaviours in receivers (Seyfarth *et al.*, 1980a, b; Struhsaker, 1967). One of these alarm call types, the ‘threat-alarm-bark’ (Struhsaker, 1967) or ‘leopard alarm’ (Seyfarth *et al.*, 1980a, b), is a loud call that only adult males produce (Dubreuil *et al.*, 2024; Price *et al.*, 2014, 2015). This vocalisation, from here on called ‘bark’, alerts group members but might also deter predators (Isbell & Bidner, 2016) by advertising the detection of a threat, which may incentivise ambush hunters to vacate an area (Adams & Kitchen, 2018; Curio, 1978; Trivers, 1971; Zuberbühler *et al.*, 1997, 1999).

The alarm call system of vervet monkeys played a pivotal role in our understanding of the cognitive processes that might underpin signal production and comprehension (Fischer & Price, 2017). However, it is less well known that male vervets sometimes utter barks in aggressive interactions (Price *et al.*, 2014; Struhsaker, 1967) and not solely in response to leopards. The acoustic structure of barks uttered during predator encounters and aggressive interactions exhibits graded variation (Price *et al.*, 2015), but it is unclear whether receivers can infer the production context from call structure alone. Playback experiments suggest that responses are highly variable (Ducheminsky *et al.*, 2014) and depend on the context at the time of the playback (Deshpande *et al.*, 2023; Price & Fischer, 2014), the signaller’s identity (Price *et al.*, 2014) and the receiver’s age (Dubreuil *et al.*, 2023). As responses to barks vary considerably (Ducheminsky *et al.*, 2014; Henzi *et al.*, 2021), receivers may not invariably infer predator presence from the occurrence of barks.

Vervet monkeys are seasonal breeders, living in multi-male, multi-female groups with female philopatry (Henzi & Lucas, 1980). Males disperse multiple times throughout their lifespan, and the time they spend in a group is highly variable, with an average residency of two mating seasons (Young *et al.*, 2019). Dispersal events occur year-round but are most common approximately 1 month before females conceive (Henzi & Lucas, 1980; Young *et al.*, 2019). Conflict intensity among males increases during the mating season, leading to severe injuries and even death (Henzi & Lucas, 1980; Minkner *et al.*, 2018). Although it remains uncertain whether male fitness is systematically tied to dominance (Minkner *et al.*, 2018), high-ranking males might reduce their rivals’ mating opportunities via coercion or be the preferred mating partners of females (Hemelrijk *et al.*, 2020; Keddy, 1986; Young *et al.*, 2017).

In this study, we aimed to enrich our understanding of the function of barks in male vervet monkeys and to gauge the potential role of barks in male–male competition. Since males occasionally bark in aggressive interactions within and between groups (Price *et al.*, 2014; Struhsaker, 1967), we hypothesised that barks serve a dual function as alarm calls and quality signals that advertise male competitive ability. If barks are honest signals of individual competitive ability, we predicted that individual calling probability and the daily number of barking events per group would increase with individual rank, the number of males per group, lower ratios of females to males, and during the mating season.

We also explored whether individual bark production was related to the number of mating seasons a male was present in a group, to assess whether past reproductive opportunity might affect bark production, since data on male reproductive success were unavailable. Lastly, we explored whether the daily number of barking events per group was related to group size because larger groups might detect more predators and could therefore exhibit more barking events per day independent of male–male competition.

## Methods

### Study Site, Study Subjects, and Data Collection

Over 24 months (2020–2022), we collected data from 45 adult males across six habituated groups of free-ranging vervet monkeys (ESM, Table S1) at the Inkawu Vervet Project in Mawana Game Reserve (28°00.327S, 031°12.348E, habitat elevation ~600–800 m), KwaZulu–Natal, South Africa. Group composition varied naturally due to birth, mortality, maturation, dispersal, and immigration (Table I). We trained all researchers to identify subjects via individually distinct morphological features, and collected data in two 8-hour shifts, starting at sunrise or 8 hours before sunset.

**Table I:** Composition of vervet groups (mean ± standard deviation) at the Inkawu Vervet Project field site in Mawana Game Reserve, KwaZulu–Natal, South Africa, 2020–2022

Group	BD	NH	LT	AK	KB	CR*
**Number of adult males**	9.7 ± 2	6.2 ± 1.5	3.4 ± 1.1	3.2 ± 1.3	2.1 ± 0.9	3.6 ± 0.7
**Number of adult females**	19.6 ± 3	11.1 ± 2.1	9.3 ± 1.4	7.4 ± 0.7	4.7 ± 1.3	7.2 ± 1.9
**Adult sex ratio**	2.1 ± 0.6	1.9 ± 0.6	3.1 ± 1.4	2.8 ± 1.3	2.6 ± 1.2	2.1 ± 0.9
**Group size**	59.2 ± 9.7	37.9 ± 5.8	26.7 ± 3.5	23.7 ± 3.4	16 ± 4.5	25.3 ± 4.8

*CR group did not contribute data in 2020 due to the COVID-19 pandemic

We collected ad libitum data on dyadic agonistic interactions between subjects, individual daily presence, and participation of adult males in barking events. We defined a barking event as the utterance of barks by one or more males within the group, and determined it to be over when no barks were produced for at least 5 minutes. We used ad-libitum sampling to record whether each male in the group uttered barks during an event (yes/no), allowing us to assess the probability that males barked relative to each other. We could not use focal sampling because it is unsuited to collect data on rare events that occur unpredictably, and we would not have been able to collect enough data on bark events. While our approach did not permit us to record the exact number of barks each male produced per event, it allowed us to record data on vocal participation (yes/no) for each male during every event.

During barking events, observers recorded the IDs of all barking males, the number of barking males that could not be identified before they ceased barking, the duration of the event, and the context of the calling bout. The context categories were: (1) terrestrial threat, including land predators and disturbances caused by other animals, (2) aerial threat, mostly raptors, (3) reptiles, mostly snakes, (4) aggressive interactions within groups, (5) new male sightings, (6) between group encounters, defined as groups ranging within 100 m of each other, (7) barks of resident males in response to distant barks from other groups outside of encounters, and (8) unknown causes, scored when all other categories were not applicable (detailed descriptions in ESM Table S7). We always scored event context as unknown when observers could not clearly identify the potential cause of barks, and never attributed contexts based on uttered call types. Since barks are audible over several hundred meters, unlike any other vocalisation in the species’ repertoire (Struhsaker, 1967), we consider it unlikely that observers missed events while they were with a group.

To estimate dominance hierarchies, we used the EloRating package version 0.46.11 (Neumann *et al.*, 2011; Neumann & Kulik, 2020), with default settings for the starting values of all individuals when they join a group (startvalue = 1000, k = 100, init = ‘average’). We used data on dyadic conflicts, with clearly identified winners and losers, from 2012 onward, with a mean of 335 interactions per group and year to determine initial rankings at the start of our study. In the 2 years of our research, we recorded 5936 decided dyadic conflicts, with a mean of 494 interactions per year and group. We estimated relative male hierarchies by ranking individual adult males according to their Elo scores. We calculated the proportion of other adult males an individual dominated on any observation day in their respective group. We chose this ranking system to obtain relative male ranks comparable between groups with different numbers of males. We began data collection on new males who immigrated into groups during our study (20/45) as soon as observers could identify them. Over the 2-year study period, 35 out of 45 males in our population dispersed, either by immigrating into a new group or by disappearing from their previous group. We determined the mating season (April to July) by assessing the distribution of conceptions in our study period, based on the known distribution of births and a 165-day gestation period (Minkner *et al.*, 2018; Young *et al.*, 2019).

### Statistical Analysis — General Procedure

We conducted all statistical analysis in R (v. 4.2.1) (R Core Team, 2023) using the lme4 package (v. 1.1–30) (Bates *et al.*, 2015) and the glmmTMB package (v. 1.1.4) (Brooks *et al.*, 2017). Since we could not always identify all callers, we compared the total dataset and the subset in which we could identify all callers by assessing the distributions of contexts, the number of callers, the number of males in the group, and the duration of barking events. Comparing the distributions of both datasets before statistical modelling was necessary to gauge the potential for sampling bias because we could only use the subset in which we identified all callers to investigate which factors affected individual calling probability.

We fitted a binomial model to test whether individual calling probability in barking events was affected by individual competitive ability and the degree of male–male competition in groups. We fitted a zero-inflated Poisson model to test whether the daily number of barking events per group was related to the degree of male–male competition in groups. Both models included all theoretically identifiable random slopes to reduce type I error rate (Barr *et al.*, 2013), and excluded correlations among random intercepts and slopes when unidentifiable (absolute correlation parameters ≥ 0.9) (Matuschek *et al.*, 2017). Visual inspection of histograms for every random intercept and slope component supported the assumption that the best linear unbiased predictors for random effects were normally distributed (Baayen, 2008). We z-transformed covariates to a mean of zero and a standard deviation of one, to ease comparability of model estimates. We dummy-coded and centred the factor mating season (yes/no) in the random effects parts of the models, using ‘outside mating season’ as the reference category (Schielzeth, 2010). We used the optimiser ‘bobyqa’ in the binomial model to assist model conversion. To rule out collinearity among predictors, we checked variance inflation factors using the R package ‘car’ (v. 3.1.0) (Fox & Weisberg, 2019), which involved running simplified general linear mixed models excluding the random effect structure and the interactions.

We checked the zero-inflated Poisson model for overdispersion to avoid potential type I errors (Gelman & Hill, 2007). To prevent ‘cryptic multiple testing’ (Forstmeier & Schielzeth, 2011), we used likelihood ratio tests to compare the full models to null models, which only included control predictors and offset terms but had the same random effects structure as the full models (Dobson & Barnett, 2018). We assessed model stability by comparing the estimates of the full model with those obtained from identical models fitted to subsets of the data that excluded the individual levels of the random effects one at a time (function provided by R. Mundry) (Nieuwenhuis *et al.*, 2012). We determined 95% confidence intervals with the ‘bootMER’ function of the ‘lme4’ package using 1000 parametric bootstraps. We derived *p*-values for individual fixed effects and interactions from likelihood ratio tests using the R function ‘drop1’ with a Chi-square test argument (Barr *et al.*, 2013). If interactions were not significant (*p* > 0.05) according to likelihood ratio tests, we fitted reduced models by removing the respective interactions as main effects without changing the random-effect structure of the models. Detailed information about model structure, random slopes, model stability and the results of likelihood ratio tests for interactions are available in the supplementary material (Table S2–S6).

### Binomial Model — Individual Calling Probability

We used a generalized linear mixed model (Baayen, 2008) with binomial error structure and logit link function to investigate which predictors influenced the probability that a male barked in a barking event. As fixed effects, we incorporated the covariates male rank (range: 0–1), number of adult males in the group (mean ± s.d. = 7.5 ± 3.3; range: 2–13), adult sex ratio (number of females per male; mean ± s.d. = 2.0 ± 0.7; range: 1–5.5), the factor mating season (yes/no), and the number of mating seasons males were present in their group (mean ± s.d. = 2.2 ± 1.4 mating seasons; range: 0–7). Collinearity among predictor variables did not pose a problem (all variance inflation factors < 1.7). We included the three-way interaction between male rank, mating season, number of adult males and the three-way interaction between male rank, mating season, and adult sex ratio while incorporating all lower order terms these encompassed. As random factors, we included individual ID, group ID, event ID, and the combination of date and group to control for potential non-independence of different events observed in the same group on the same day. We excluded all events in which unknown males produced barks from the analysis, since the barking activity of unknown callers would introduce uncertainty into the binomial response ‘male produced barks (yes/no)’. We also excluded events if the group they occurred in only had a single resident adult male at the time of the event, since the predictor relative male rank is not defined in single-male groups.

### Poisson Model — Number of Barking Events Per Observation Day

To assess whether group composition and the mating season affected the number of barking events recorded per day, we used a generalized linear mixed model with a Poisson error structure and a log-link function accounting for zero inflation (Brooks *et al.*, 2017). We included the covariates number of adult males (mean ± = 5.6 s.d. ± 3.1; range: 1–13), adult sex ratio (mean ± s.d. = 2.4 ± 1.1; range: 0.7–12) and the factor mating season (yes/no) as fixed effects. Further, we added the interaction between mating season and number of adult males as well as the interaction between mating season and adult sex ratio. As a control predictor, we included the covariate group size (mean ± s.d. = 36.6 ± 15.8; range: 5–77). To account for observation effort, we added the log-transformed observation time of every observation day as an offset term (mean ± s.d. = 6.9 ± 2.1; range: 2.5–14), and excluded days with missing observation times from the analysis. We included group ID as a random factor. An initial Poisson model revealed that the observed number of zeros was higher than expected, given the model. To account for zero inflation, we incorporated a random intercept in the zero-inflation part of the model. We ruled out overdispersion (dispersion parameter = 1.08). We excluded collinearity among predictor variables for ‘mating season’ and ‘sex ratio’ with variance inflation factors < 1.9. We could not exclude potential collinearity for the predictor ‘number of adult males’ and the control predictor ‘group size’ with variance inflation factors < 6.7 and < 5.1 respectively. Since larger groups typically contain more adult males, a positive relationship between the number of adult males in a group and group size can be expected. Because group composition fluctuated during the study, we decided that controlling for group size was required and did not change the model structure. To assess whether collinearity masked potential effects, we ran two additional reduced models excluding either ‘group size’ or ‘number of adult males’ respectively.

## Ethical Approval

This research adhered to the Association for the Study of Animal Behaviour Guidelines for the Use of Animals in Research (Behaviour, 2018), the regulations set by the local authority, Ezemvelo KZN Wildlife, and the Animal Care Committee at the German Primate Center. We further obtained permission from the van der Walt family, the owners of the Mawana Game Reserve. The authors declare that they have no conflict of interest.

### Data Availability

The datasets and scripts generated during and/or analysed during the current study are available in the OSF repository: 10.17605/OSF.IO/2W7ZH.

## Results

### Binomial Model – Individual Calling Probability

Over 24 months, we recorded 886 barking events from six groups containing 45 adult males. Maps showing the GPS coordinates of events and estimated audible distances of barks are available in the electronic supplementary material (ESM, Fig. S1–S2), in addition to distributions of diurnal and seasonal variation in bark production (Fig. S3–S4), and the monthly distribution of births in our study time (Fig. S5).

**Fig. 1:**
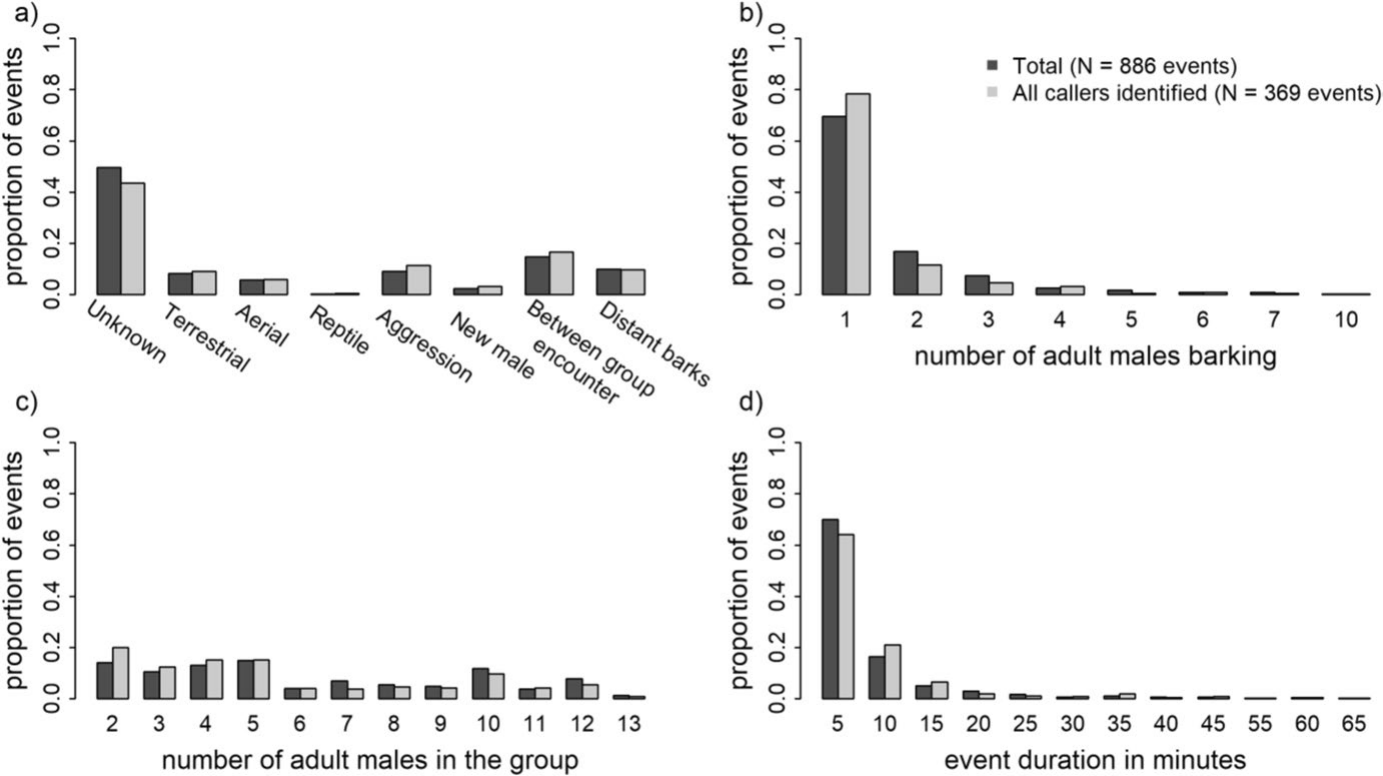
Distributions of barking events recorded for vervet monkeys at the Inkawu Vervet Project field site, Mawana Game Reserve, KwaZulu–Natal, South Africa, from 2020 to 2022. **a** Event contexts (descriptions in ESM Table S7). **b** Number of adult males barking. **c** Number of adult males present in the group. **d** Event duration in 5-minute bins (5 = 0 to 5 min; 10 = 5 to 10 min, etc.).

After excluding all events with unknown male callers and all events in single male groups, the sample for the model comprised 369 events, yielding 2055 data points (with 524 barking participations) from 45 adult males in six groups over 291 group observation days. Barking occurred in different predator contexts, during aggressive interactions within and between groups, when new males were seen following the group and in response to distant barks from other groups (Fig. 1a). Observers could not clearly assign most events to any specific context and therefore classified them as ‘unknown’.

Visual assessment suggested that the distributions of the total dataset (*n* = 886 events) and the subset of with only known callers (*n* = 369 events) are similar (Fig. 1), but we cannot rule out sampling bias entirely. The majority of events only had a single caller (Fig. 1b). Most events lasted no longer than 5 minutes, but we also recorded durations of over 1 hour (Fig. 1d). We found no peaks in event frequency around sunrise or sunset (Fig. S3).

**Fig. 2:**
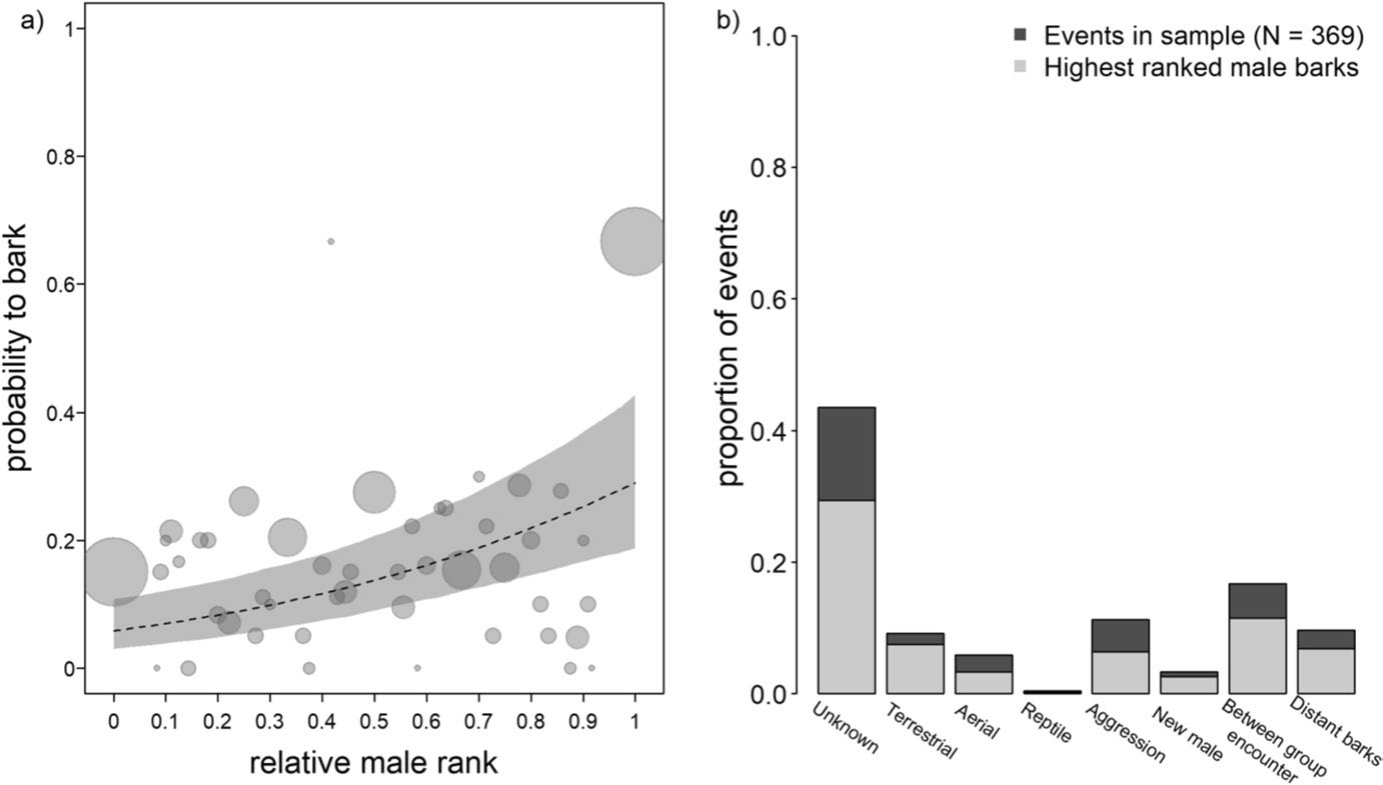
**a** Probability of barking in relation to individual rank (proportion of other males dominated in the group) for vervet monkeys at the Inkawu Vervet Project field site, in Mawana Game Reserve, KwaZulu–Natal, South Africa, from 2020 to 2022. The plot shows the mean barking probability for each rank value, with the *circle area* proportional to the frequency at which a given rank value occurred. The *dashed line* depicts the fitted model, and the *grey shaded area* shows the bootstrapped 95% confidence intervals, with all other predictors being at their mean for covariates and the factor mating season being dummy-coded and centred. Of the 45 adult males in the data, 23 individuals held the highest rank at some point during the study. **b** Distribution of the contexts of analysed barking events.

Comparing the full and null model showed that the probability that an individual male barked in an event was significantly linked to the fixed effects of the model (*χ2* = 25.326, *df* = 12, *p* = 0.013). Since likelihood ratio tests indicated that the three-way and two-way interactions were not significant (ESM, Table S2–S3), we removed them and fitted a reduced model. The reduced model revealed no obvious effects of adult sex ratio, mating season, or the number of mating seasons present (Table II). Higher-ranking individuals were likelier to bark (GLMM: *n* = 2055, *p* = 0.002, Fig. 2a and Table II). The positive effect of rank on calling probability was moderate overall, except for the highest-ranked males in a group, who showed very high calling probability (Fig. 2a). Visual inspection of the model (Fig. 2a) indicated a poor model fit, suggesting that the behaviour of the highest ranked males was driving the positive effect of rank. We, therefore, refitted the model excluding the highest-ranked males (rank = 1; 23 of 45 males temporarily held the highest rank). While the estimate for rank was still positive, visual inspection of the refitted model supported the assumption that the effect of rank was mainly driven by barking activity from the highest-ranked males in the group (ESM, Fig. S6).

**Table II:** Results of a GLMM examining predictors of the probability that adult male vervet monkeys barked in a barking event (*n* = 45) at the Inkawu Vervet Project field site in Mawana Game Reserve, KwaZulu–Natal, South Africa, from 2020 to 2022

Term	Estimate	SE	Lower CI	Upper CI	*χ2*	*df*	*p*
**Intercept**	−1.767	0.266	−2.263	−1.165			*
**Rank**	0.667	0.144	0.363	0.941	9.589	1	0.002
**Number of adult males**	−0.919	0.238	−1.399	−0.421	10.479	1	0.001
**Adult sex ratio**	0.022	0.139	−0.272	0.315	0.024	1	0.876
**Mating season (Yes)**	−0.133	0.238	−0.617	0.341	0.325	1	0.568
**Number of mating seasons present**	0.268	0.184	−0.110	0.685	2.021	1	0.155

* Not shown due to limited interpretability

As the context of most events was unknown, we could not include context in the model. To assess whether a specific context drove the effect of rank, we plotted the distribution of contexts combined with the participation rate of the highest-ranked male (Fig. 2b), which suggested that the effect was not context-specific. We also plotted calling probability against rank across all events, including those with unknown callers, which indicated no sampling bias (Fig. S7). The high number of males that, at some point, held the highest rank in their group (23 of 45) can be attributed to natural dispersal events (35 of 45 males dispersed, ESM Table S1).

**Fig. 3:**
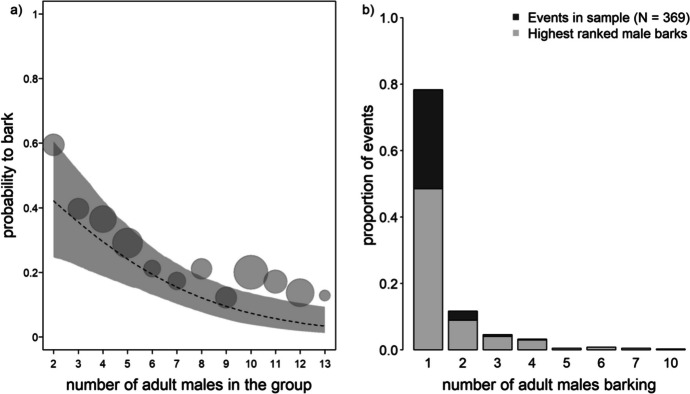
**a** Probability of barking for any given male in relation to the number of males in the group for vervet monkeys at the Inkawu Vervet Project field site in Mawana Game Reserve, KwaZulu–Natal, South Africa, from 2020 to 2022. The *circle area* is proportional to the frequency at which a given number of males occurred. The *dashed line* depicts the fitted model and the *grey shaded area* shows the bootstrapped 95% confidence intervals, with all other predictors at their mean or dummy coded and centred. **b** Distribution of the total number of callers across analysed barking events.

In addition to a positive effect of the highest rank on calling probability, the model revealed that a higher number of adult males in the group was associated with decreased individual barking probability (GLMM: *n* = 2055, *p* = 0.001, Fig. 3a and Table II). The distribution of the number of calling males showed that in most events, only a single male called (Fig. 1b and 3b). The highest-ranked males were responsible for 62% of single male calling events. They also barked in 84% of all events with more than one caller (Fig. 3b).

### Poisson Model — Number of Barking Events Per Observation Day

**Fig. 4:**
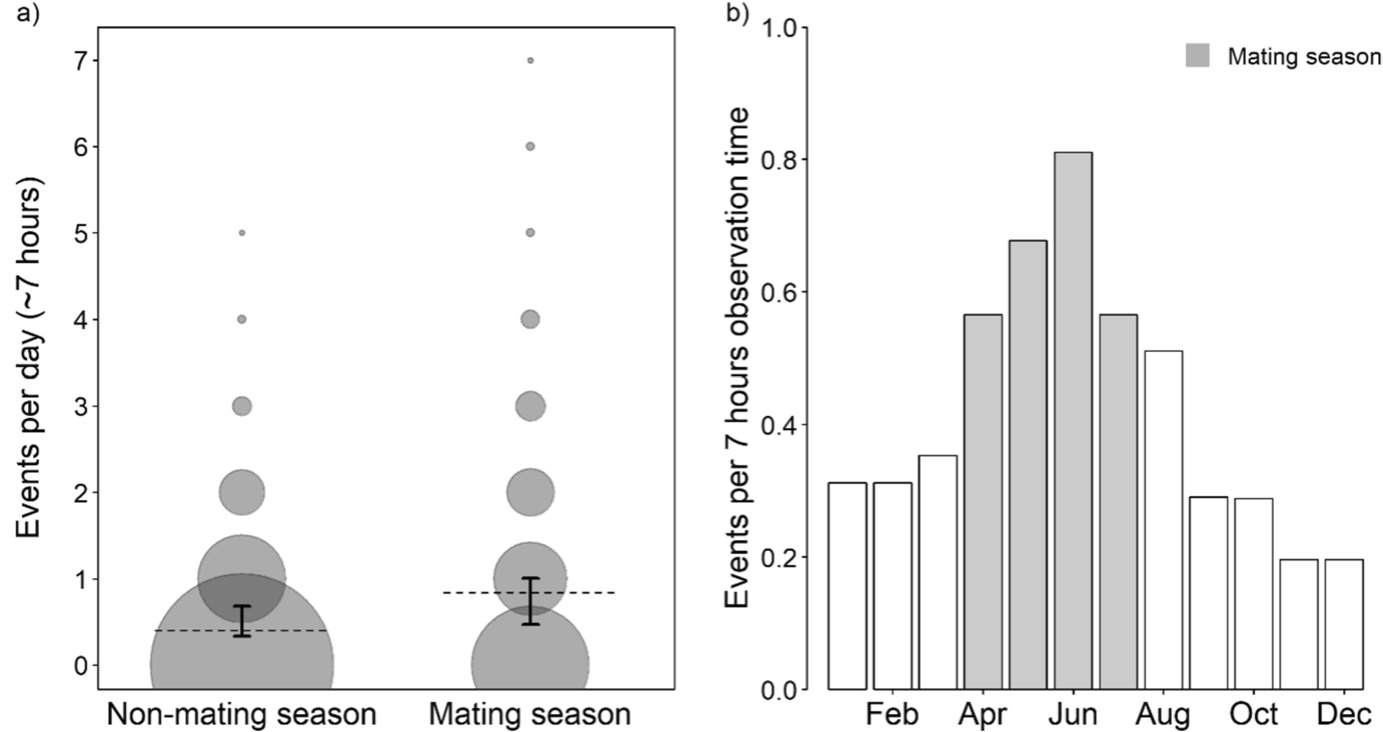
**a** The effect of the mating season on the number of barking events recorded per observation day from groups of vervet monkeys at the Inkawu Vervet Project field site in Mawana Game Reserve, KwaZulu–Natal, South Africa, from 2020 to 2022, with a mean observation time of approximately 7 hours per observation day. The *circle area* is proportional to the frequency of the recorded events. The *dashed grey line* depicts the fitted model, and *black error bars* indicate the bootstrapped 95% confidence intervals, with all other predictors being at their mean. **b** The monthly number of events per 7 hours of observation time (*n* = 1915 group observation days from 2020 to 2022).

After excluding days with missing observation times, the sample for analysis comprised 1915 group observation days, with 13,243 observation hours, containing 821 barking events from six groups. The fixed effects significantly impacted the number of group-level barking events per day (*χ2* = 20.387, *df* = 5, *p* = 0.001). Subsequent likelihood ratio tests indicated that none of the interactions were significant (ESM, Table S4). The reduced model revealed that the number of barking events per day increased during the mating season (GLMM: *n* = 1915, *p* < 0.001, Fig. 4a, b and Table III; see ESM Fig. S4–S5 for the monthly distribution of barking events and conceptions). While barking events were generally rare, they occurred about twice as often during the mating season, with one barking event approximately every 11 hours of observation time compared to every 23 hours outside the mating season. Group size, adult sex ratio, and the number of adult males had no statistically significant effects on the number of barking events per day (Table III). Additional reduced models excluding either group size or number of adult males confirmed that neither of the two predictors had a statistically significant effect in the absence of the other, suggesting collinearity did not mask potential effects (Table S5 and S6).

**Table III:** Results of a GLMM examining predictors of the number of barking events per group observation day, recorded from groups (*n* = 6) of vervet monkeys at the Inkawu Vervet Project field site in Mawana Game Reserve, KwaZulu–Natal, South Africa, from 2020 to 2022

Term	Estimate	SE	Lower CI	Upper CI	*χ2*	*df*	*p*
**Intercept**	−2.658	0.095	−2.876	−2.490			*
**Number of adult males**	0.187	0.117	−0.068	0.402	2.353	1	0.125
**Adult sex ratio**	0.042	0.084	−0.139	0.186	0.274	1	0.601
**Mating season (Yes)**	0.742	0.098	0.532	0.930	14.798	1	<0.001
**Group size**	−0.109	0.105	−0.325	0.108	1.087	1	0.297
**Zero-inflation-intercept**	−0.555	0.145	−0.886	−0.303			*

* Not shown due to limited interpretability

## Discussion

Male vervet monkeys uttered barks in response to terrestrial and aerial predators, during aggressive interactions within and between groups, upon encountering new males, and occasionally when distant barking from neighbouring groups was audible. Capturing context unambiguously was impossible in about half of the events, but aggressive interactions accounted for a quarter of observations, confirming reports that barks also occur in conflicts (Price & Fischer, 2014; Struhsaker, 1967).

The probability that males barked during a barking event varied with individual rank and the number of males in a group. Only the highest-ranked males exhibited high calling probabilities, and were thus responsible for the observed positive effect of rank. Frequent bark production in vervet monkeys, therefore, might advertise high caller rank, as is also the case for the loud calling behaviour of chacma baboons, *Papio ursinus*, and crested macaques, *Macaca nigra* (Fischer *et al.*, 2004; Kitchen *et al.*, 2003; Neumann *et al.*, 2010).

Most barking events only had a single caller, explaining why individual calling probability decreased with a growing number of males in a group. The highest-ranking males were thus not only the most likely individuals to call in any event, but were frequently the only callers. We found no evidence for a relationship between individual barking probability and a group´s adult sex ratio, the mating season, and the number of mating seasons in which an individual was present. Therefore, neither male–male within-group competition nor past reproductive opportunities of males appeared to be related to individual barking probability. Instead, individual baseline calling probability was determined by how many males were in the group, while only high rank seemed to increase calling probability markedly.

Observational studies of the Amboseli vervet population in Kenya, examining which individuals were the first to utter alarm calls, reported that call frequency increased with caller rank (Cheney & Seyfarth, 1981; Seyfarth & Cheney, 1985). A similar report from the Windy Ridge population in South Africa suggested that the highest-ranked male uttered alarms more frequently than other males (Baldellou & Henzi, 1992). Comparing our data to these studies is not straightforward, since they focused on which individual called first and pooled the three alarm call types of vervet monkeys. Our data are consistent with the idea that rank predicts call rates. However, since barks often occurred in aggressive interactions and high-ranking males frequently barked alone, our results support the notion that this vocalisation might play a role during male–male competition, in addition to deterring predators (Gautier & Gautier-Hion, 1977; Price *et al.*, 2014, 2015; Struhsaker, 1967).

At the group level, the daily number of barking events was unaffected by group size, the adult sex ratio, and the number of males present, but increased notably during the mating season. The temporal distribution of births during our study suggested that most females conceived between May and June, coinciding with the highest numbers of monthly barking events. Apparently, male vervet monkeys increase their loud calling frequency around the time of ovulation, similar to other catarrhine primate species (Fuller & Cords, 2017, 2020; Harris, 2006). While female vervet monkeys lack obvious sexual signals that advertise their receptivity (Andelman, 1987), males might respond to the seasonal increase in mating behaviour, which cannot be concealed (Young *et al.*, 2019). Alternatively, the rise in bark rates might be a consequence of seasonally increasing male–male aggression and vigilance (Baldellou & Henzi, 1992; Henzi *et al.*, 2021; Minkner *et al.*, 2018) or the influx of new males into groups (Hemelrijk *et al.*, 2020; Henzi & Lucas, 1980).

Our data are consistent with the hypothesis that barks serve as quality signals that advertise male competitive ability (Price *et al.*, 2014). Zuberbühler (2002) suggested that alarm calls of male forest guenons initially evolved to serve as predator deterrents and were subsequently modified by sexual selection to indicate signaller quality. As in other primate species where male loud call use varies with signaller rank, condition, or exhaustion after male–male conflicts (Benítez *et al.*, 2016; Fischer *et al.*, 2004; Harris, 2006; Kitchen *et al.*, 2003; Neumann *et al.*, 2010; Teichroeb & Sicotte, 2010), vervet monkey barks might serve as ‘quality handicaps’ during male–male competition. According to this hypothesis, lower-quality males are assumed to lack the stamina for regular barking activity. Since we could not quantify the number of calls that individuals produced during call bouts, evidence for a relationship between rank and stamina remains limited (Cheney & Seyfarth, 1981).

As most events were short and just had one caller, opportunities for energetically costly signalling contests between males appeared rare. Barks might therefore also serve as conventional signals that primarily indicate male motivation to compete (Enquist, 1985; Guilford & Dawkins, 1995; Vehrencamp, 2000). Conventional signals can remain honest without relevant signal production costs due to a receiver retaliation rule (Vehrencamp, 2000). Since low-quality signallers are at a disadvantage in the event that receivers attack them in response to their signal, the costs of signalling are disproportionately higher for low-quality individuals. If dominant males punish subordinates that bark outside of predator encounters, one would expect that bark production is suppressed in all but the highest-ranked individuals. In this scenario, frequent calling would follow acquiring high rank rather than leading to it.

Although barking during between-group encounters could assist in territory defence, many males do not reside in the same group for more than two mating seasons (Young *et al.*, 2019). Philopatric females are therefore expected to benefit more from males who bark to defend their territory than the males themselves, unless male investment in territory defence is favoured via inter-sexual selection (van Schaik *et al.*, 2022). Alternatively, males might also bark in encounters to repel rivals, or to assess their own barking performance relative to that of males from other groups, possibly gauging the risk of dispersal to themselves.

In summary, we found that vervet monkey bark use is not restricted to predator contexts, exhibits seasonal variation, and is tied to signaller rank. We suggest that future studies examine whether call rates and the acoustic structure of barks are related to caller rank and age. Evidence that the relationship between vocal behaviour and individual rank also extends to increased reproductive success (Snowdon, 2004) would support the hypothesis that barks also serve as quality signals, in addition to their alarm call function.

## Supplementary Information

Below is the link to the electronic supplementary material.

Supplementary file1 (1.30 MB)

## Data Availability

The datasets and scripts generated during and/or analyzed during the current study are available in the OSF repository, 10.17605/OSF.IO/2W7ZH.
