# Supplementary material for: Loud Call Production in Male Vervet Monkeys (Chlorocebus pygerythrus) Varies with Season and Signaller Rank
Source: Int J Primatol. 2025 Jan 23;46(2):538–55. doi: 10.1007/s10764-024-00475-x (PMC12045810; doi:10.1007/s10764-024-00475-x)
Supplement: Supplementary file 1 — Supplementary file1 (1.30 MB) [file 10764_2024_475_MOESM1_ESM.pdf]

Electronic supplementary material for

Loud call production in male vervet monkeys (*Chlorocebus pygerythrus*) varies with season and signaller rank

Supplementary figures and tables

Barking events were recorded throughout the home ranges of all six groups (Fig. S1). When a barking event from one group was audible to a neighbouring group, outside of between group encounters, we occasionally estimated the transmission range of barks, by comparing GPS positions of the respective groups (Fig. S2). While we only made few opportunistic recordings and could not account for weather, vegetation or caller elevation, we estimated barks to be audible over 900 m in some cases (Fig. S2).

There was no discernible increase in bark event frequency at the time of sunrise or sunset (Fig. S3). The distribution of the number of barking events per month indicated an increase in monthly event frequencies during the mating season (Fig. S4). The distribution of births during the study period suggested that most conceptions occurred from May to June, and only one apparently outside the mating season (Fig. S5). Barking activity was highest around the time that most females conceived (Fig. S4 and S5).

Since visual inspection of the first model (Fig. 2a) suggested that the effect of rank on barking probability appeared to depend strongly on the behaviour of the highest-ranked males, we fitted the model again but excluded the highest ranked males (Fig. S6). The model without data from the highest-ranked males corroborated the assumption that the effect of rank on barking probability was strongly driven by the highest-ranked individuals (Fig. S6). Plotting calling probability against male rank across all events, including those with unknown callers, suggested no sampling bias (Fig. S7, for comparison see Fig. 2a and S6).

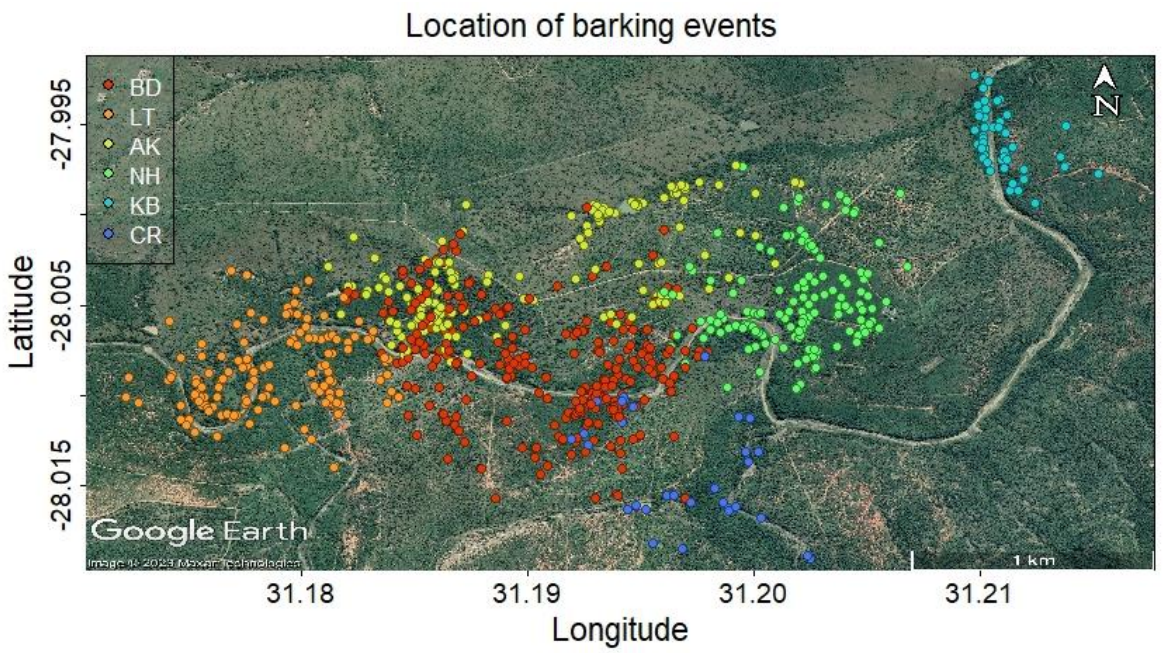

Fig. S1 Location of barking events recorded from the six groups with available GPS data (N=840). Data collection on the CR group was interrupted in 2020 due to the COVID-19 pandemic, resulting in a lower number of observed barking events.

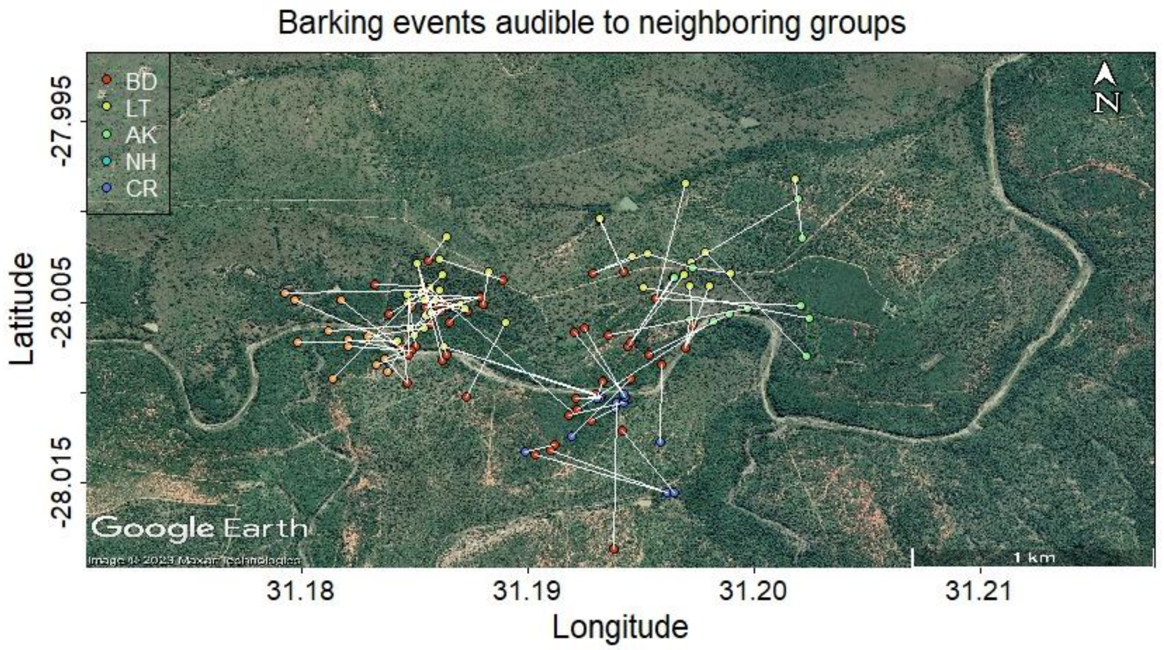

Fig. S2 Barking event location relative to the location of a neighbouring group, at the time of the event, indicated by white lines. Recordings were made opportunistically when observers heard barks from another group outside of encounters and could confirm the barking group's identity via radio contact with observers in the barking group (N=93).

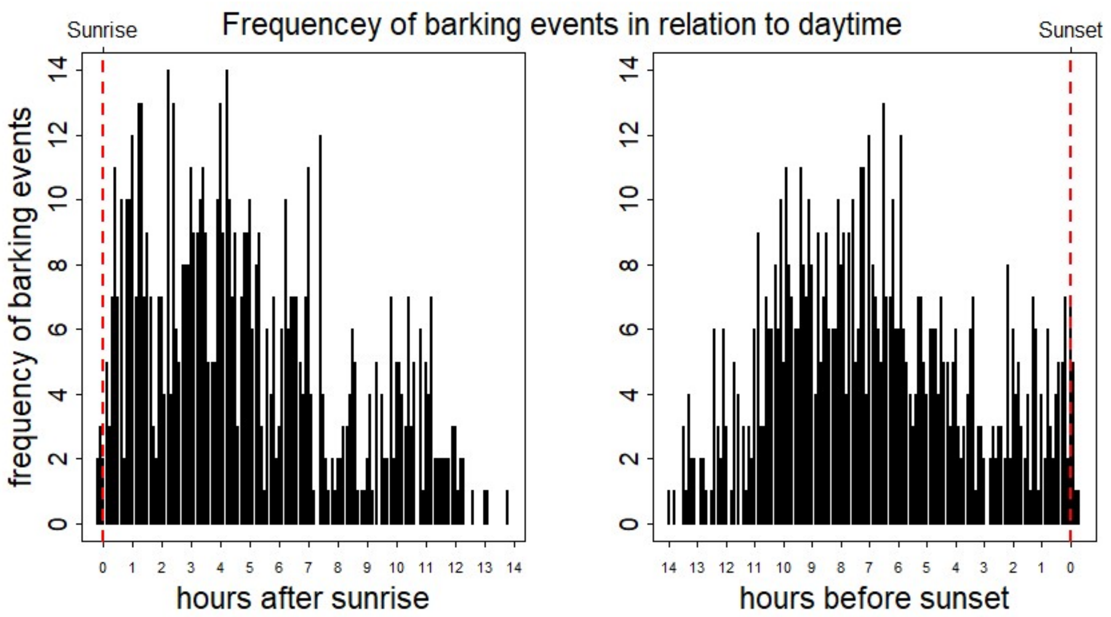

Fig. S3 Frequency of barking events in relation to relative daytime. Both plots depict the same data, shown relative to sunrise and sunset due to seasonal variation in day length.

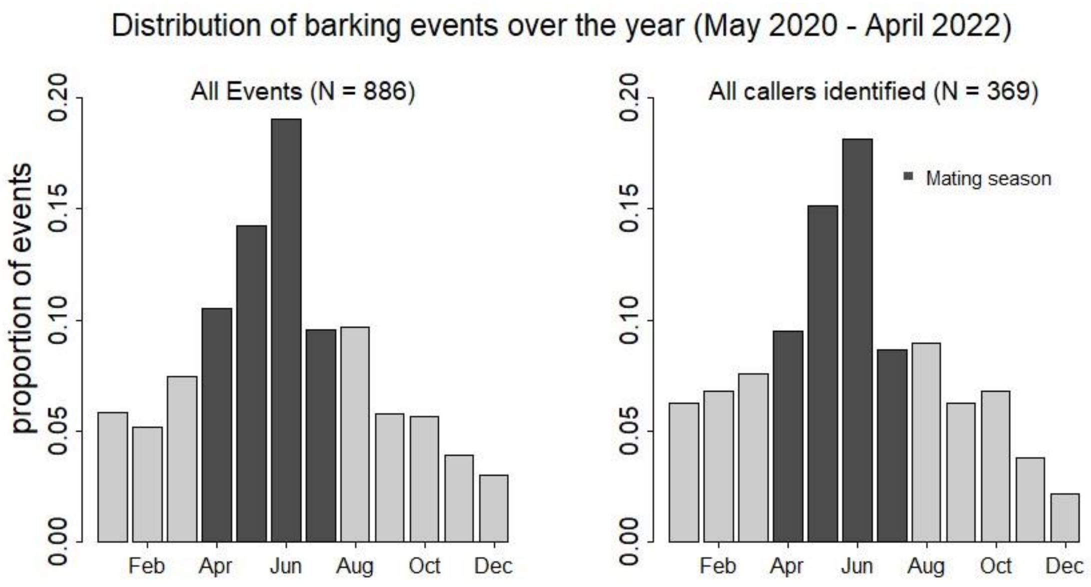

Fig. S4 Proportion of calling events per month with mating season indicated.

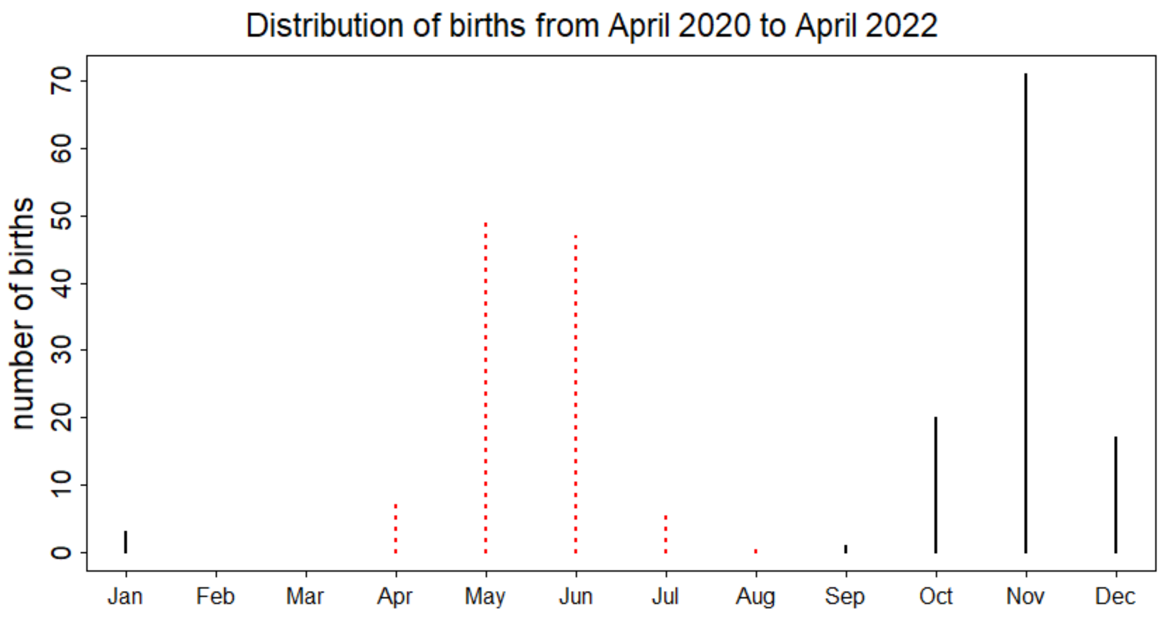

Fig. S5 Total monthly number of births (black lines) and dated back times of conception (dashed red lines) in all six groups for the two year study period (N=112 births). The majority of births occurred in November. With a gestation period of 165 days, most conceptions occurred in May and June.

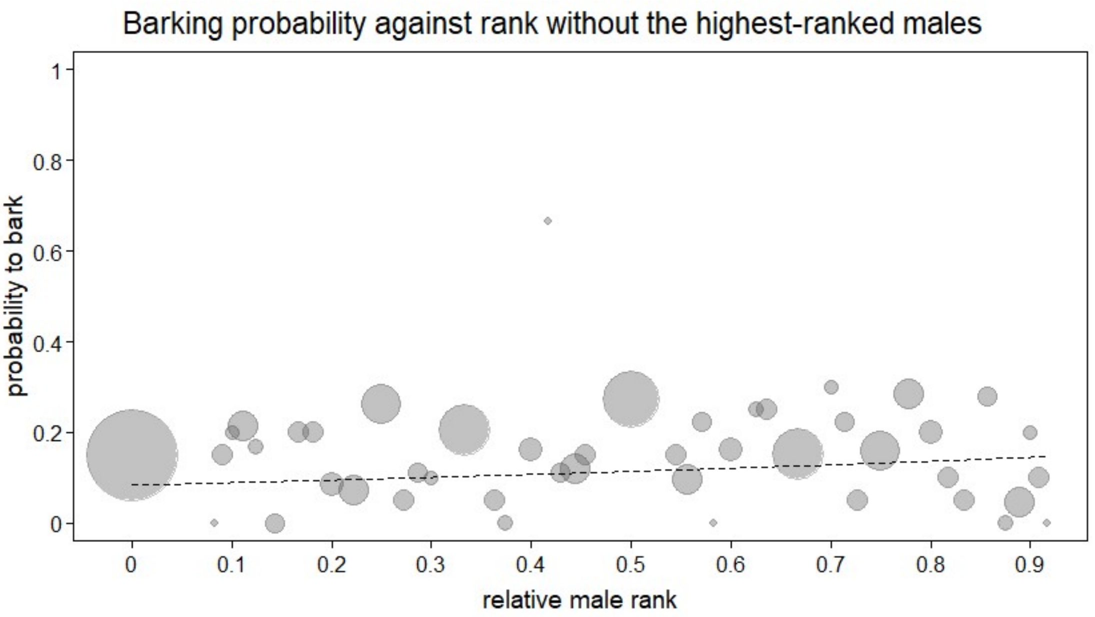

Fig. S6 The effect of individual rank on barking probability excluding the highest ranked males (Rank = 1; 23 of 45 males temporarily held the highest rank, N = 369 events). The effect of rank was likely driven by the highest ranked males (Estimate = 0.202, Std. error = 0.166, z value = 1.215)

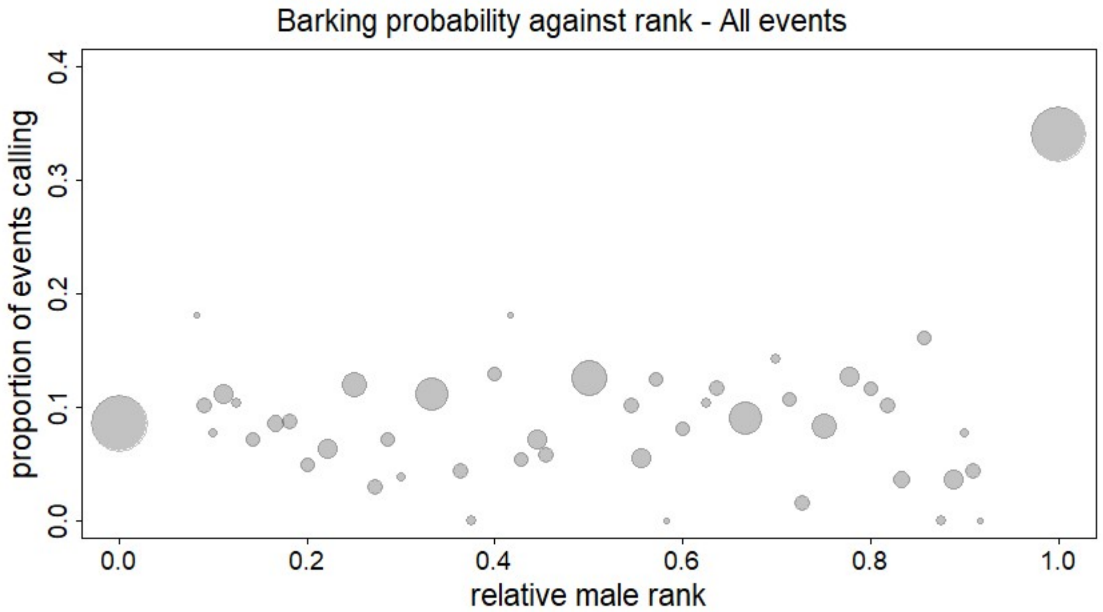

Fig. S7 Barking probability against male rank across all events (N = 886 events), including events with unknown callers.

Table S1: Individual information. Number of events a subject was present, barking and highest-ranked male. Proportion of events (Prop.) a subject was barking and highest-ranked male. Mean and standard deviation of subject rank. Range of the number of mating seasons (M.S.) a subject was present in a group. Table sorted by prop. alpha in descending order.

Subject ID	Events present	Events Barking	Prop. barking	Events Alpha	Prop. alpha	Rank mean	Rank s.d.	M.S. range
**10**	8	5	0.62	8	1.00	1.00	0.00	1-1
**15**	12	9	0.75	12	1.00	1.00	0.00	2-2
**27**	17	16	0.94	17	1.00	1.00	0.00	2-2
**29**	90	48	0.53	82	0.91	0.96	0.14	2-4
**50**	107	97	0.91	83	0.78	0.83	0.33	1-2
**14**	63	40	0.63	45	0.71	0.82	0.31	2-3
**3**	17	4	0.24	10	0.59	0.78	0.27	2-2
**42**	5	1	0.20	2	0.40	0.92	0.10	5-5
**18**	28	10	0.36	11	0.39	0.87	0.12	1-7
**46**	54	20	0.37	21	0.39	0.77	0.26	1-3
**45**	61	23	0.38	22	0.36	0.56	0.38	1-2
**40**	23	13	0.57	8	0.35	0.48	0.44	1-2
**11**	32	1	0.03	8	0.25	0.63	0.29	1-3
**23**	31	2	0.06	6	0.19	0.78	0.11	7-7
**26**	28	11	0.39	5	0.18	0.25	0.40	1-2
**37**	48	18	0.38	8	0.17	0.55	0.38	1-2
**34**	28	8	0.29	4	0.14	0.38	0.35	0-1
**33**	99	8	0.08	9	0.09	0.24	0.37	0-2
**44**	41	14	0.34	2	0.05	0.47	0.20	1-3
**28**	88	16	0.18	3	0.03	0.77	0.16	4-5
**38**	60	4	0.07	1	0.02	0.24	0.19	1-1
**31**	75	5	0.07	1	0.01	0.75	0.16	3-4
**32**	94	7	0.07	1	0.01	0.50	0.24	2-4
**1**	39	4	0.10	0	0.00	0.49	0.16	1-1
**2**	10	5	0.50	0	0.00	0.18	0.25	3-3

**Subject ID**	Events present	Events Barking	Prop. barking	Events Alpha	Prop. alpha	Rank mean	Rank s.d.	M.S. range
**4**	34	13	0.38	0	0.00	0.57	0.13	4-4
**6**	58	6	0.10	0	0.00	0.56	0.18	1-2
**7**	24	2	0.08	0	0.00	0.66	0.08	7-7
**8**	17	1	0.06	0	0.00	0.83	0.04	3-3
**9**	95	18	0.19	0	0.00	0.15	0.13	2-4
**12**	93	25	0.27	0	0.00	0.57	0.15	2-4
**16**	17	0	0.00	0	0.00	0.01	0.06	1-1
**17**	90	17	0.19	0	0.00	0.38	0.21	1-3
**19**	13	0	0.00	0	0.00	0.45	0.09	2-2
**20**	78	9	0.12	0	0.00	0.23	0.12	1-2
**21**	39	7	0.18	0	0.00	0.17	0.13	1-1
**24**	8	1	0.12	0	0.00	0.18	0.10	0-1
**25**	9	6	0.67	0	0.00	0.59	0.15	2-2
**30**	34	3	0.09	0	0.00	0.08	0.21	1-1
**36**	43	4	0.09	0	0.00	0.10	0.16	1-3
**39**	93	2	0.02	0	0.00	0.04	0.10	2-4
**41**	16	7	0.44	0	0.00	0.00	0.00	1-1
**43**	95	4	0.04	0	0.00	0.56	0.19	2-4
**47**	29	2	0.07	0	0.00	0.28	0.15	0-1
**49**	12	8	0.67	0	0.00	0.19	0.22	6-6

Supplementary tables

Table S2 Model 1 – Individual calling probability in barking events – Full model

Model formula:

glmer(Bark ~ z.Rank*z.Males*MatingSeason + z.Rank*z.SexRatio*MatingSeason +z.Tenure+

(1+ z.Rank*z.Males*MatingSeason.1 + z.Rank*z.SexRatio*MatingSeason.1 +z.Tenure ||Individual)+

(1+ z.Rank*z.Males*MatingSeason.1 + z.Rank*z.SexRatio*MatingSeason.1 +z.Tenure ||Group)+

(1 + z.Rank |Event_ID)+

(1 + z.Rank ||Date.in.group),

data = t.data, family=binomial, control=glmerControl(optimizer="bobyqa", optCtrl = list(maxfun=1000000)))

Results of binomial model on barking probability. Shown are model estimates, standard errors, confidence intervals (CI), the test results obtained from likelihood ratio tests (*χ2*, *df*, *P*) and the range of estimates obtained when dropping levels of grouping factors one at a time (Min, Max). All covariates (Rank, Males, SexRatio and TenureMS) were z-transformed to a mean of 0 and a standard deviation of 1. The factor MatingSeason was dummy coded and centered in the random effects part, with the reference category set to outside MatingSeason.

Term	Estimate	Std. Error	2.5% CI	97.5% CI	*χ2*	*df*	*P*	Min	Max
Intercept	-1.818	0.261	-2.28	-1.325	(-)	(-)	(-)	-1.934	-1.5
z.Rank	0.75	0.234	0.306	1.166	(-)	(-)	(-)	0.453	1.61
z.Males	-1.063	0.268	-1.576	-0.504	(-)	(-)	(-)	-1.285	-0.945
MatingSeasonY	-0.047	0.207	-0.463	0.367	(-)	(-)	(-)	-1.082	0.037
z.SexRatio	0.028	0.17	-0.312	0.343	(-)	(-)	(-)	-0.07	0.109
z.Tenure	0.267	0.188	-0.108	0.639	1.91	1	0.167	0.101	0.711
z.Rank * z.Males	0.064	0.236	-0.4	0.508	(-)	(-)	(-)	-0.094	0.979
z.Rank * MatingSeasonY	-0.168	0.329	-0.711	0.466	(-)	(-)	(-)	-0.5	0.024
z.Males * MatingSeasonY	0.286	0.227	-0.192	0.768	(-)	(-)	(-)	-0.743	0.424
z.Rank * z.SexRatio	0.1	0.145	-0.161	0.396	(-)	(-)	(-)	-0.084	0.295
MatingSeasonY * z.SexRatio	-0.098	0.237	-0.546	0.403	(-)	(-)	(-)	-0.332	0.042
z.Rank * MatingSeasonY * z.Males	-0.073	0.332	-0.657	0.515	0.048	1	0.827	-0.475	0.101
z.Rank * MatingSeasonY * z.SexRatio	-0.11	0.222	-0.552	0.29	0.25	1	0.616	-0.267	0.05

Model sample: N = 2055, Distribution of response: Bark (Yes) = 524, Bark (No) = 1531

Grouping factors: Individual (N=45), Group (N=6), Event ID (N=369), Date in Group (N=291)

Table S3 Model 1 – Individual calling probability in barking events – Results of likelihood ratio tests assessing the two-way interactions

**Term**	***χ2***	***df***	***P***
z.TenureMS	1.871	1	0.171
z.Rank * z.Males	0.025	1	0.874
z.Rank * MatingSeason	0.21	1	0.647
z.Males * MatingSeason	1.101	1	0.294
z.Rank * z. SexRatio	0.244	1	0.622
MatingSeason * z. SexRatio	0.223	1	0.637

Table S4 Model 2 – Bark events per day – Full model

Model formula:

glmmTMB(BarkEvents ~ MatingSeason * (z.Males + z.SexRatio) + z.GroupSize +
offset(log.TimeSpent) +
(1 + MatingSeason.1 * (z.Males + z. SexRatio) + z. GroupSize ||Group),
data = t.data, family=poisson, ziformula = ~1)

Results of the Poisson model on the number of barking events per day. Shown are model estimates, standard errors, confidence intervals (CI), the test results obtained from likelihood ratio tests (*χ2*, *df*, *P*) and the range of estimates obtained when dropping levels of grouping factors one at a time (Min, Max). All covariates (Males, SexRatio, GroupSize) were z-transformed to a mean of 0 and a standard deviation of 1. The factor MatingSeason was dummy coded and centered in the random effects part, with the reference category set to outside MatingSeason.

Term	Estimate	Std. Error	2.5% CI	97.5% CI	*χ2*	*df*	*P*	Min	Max
Intercept	-2.647	0.089	-2.843	-2.489	(-)	(-)	(-)	-2.814	-2.572
MatingSeasonY	0.712	0.090	0.531	0.886	(-)	(-)	(-)	0.65	0.884
z.Males	0.255	0.145	-0.002	0.503	(-)	(-)	(-)	-0.121	0.309
z.SexRatio	0.169	0.138	-0.032	0.369	(-)	(-)	(-)	0.053	0.268
z. GroupSize	-0.126	0.11	-0.341	0.083	1.342	1	0.247	-0.185	-0.042
MatingSeasonY * z.Males	-0.091	0.097	-0.283	0.096	0.957	1	0.328	-0.161	0.173
MatingSeasonY * z.SexRatio	-0.243	0.146	-0.493	0	2.4	1	0.121	-0.36	-0.105
Zi@Intercept	-0.55	0.145	-0.881	-0.325	(-)	(-)	(-)	-0.644	-0.480

Model sample: N = 1915, Grouping factors: Group (N=6), Dispersion parameter: 1.08

Table S5 Bark events per day – reduced model without the predictor group size

The predictor ‘males’ does not have an effect in the absence of the predictor ‘group size’. Collinearity among the two predictors did not mask potential effects.

Term	Estimate	SE	*χ2*	*df*	*P*
**Intercept**	-2.664	0.091			*
**MatingSeasonY**	0.755	0.096	15.213	1	<0.001
**SexRatio**	0	0.084	0	1	1
**Males**	0.086	0.07	1.408	1	0.235
**Zero-inflation-Intercept**	-0.558	0.146			*

* Not shown due to limited interpretability.

Table S6 Bark events per day – reduced model without the predictor males

The predictor ‘group size’ does not have an effect in the absence of the predictor ‘males’. Collinearity among the two predictors did not mask potential effects.

Term	Estimate	SE	*χ2*	*df*	*P*
**Intercept**	-2.671	0.093			*
**MatingSeasonY**	0.751	0.096	15.089	1	<0.001
**SexRatio**	-0.047	0.081	0.328	1	0.567
**GroupSize**	0.028	0.072	0.141	1	0.707
**Zero-inflation-Intercept**	-0.558	0.146			*

* Not shown due to limited interpretability.

Table S7 Descriptions of context categories that adult males responded to by producing barks. Researchers could assign multiple contexts for the same event if applicable.

Context	Description
**Aerial**	Any aerial object, including bird species (predominantly raptors), occasionally helicopters, and small airplanes.
**Aggression**	Within group conflicts.
**Between group encounter (BGE)**	Encounters of one or more vervet monkey groups, defined as ranging within 100 meter of each another.
**Distant barks**	Barks from other groups faintly audible in the distance and outside of encounters. Note that in this dataset such events were only considered, if one or more males in the observed group responded to distant barks by producing barks themselves. Distant barking events without vocal responses from the observed group were not considered.
**Reptile**	Encounters with reptile species including mostly pythons, black mambas, spitting cobras, puff adders, spotted bush snakes and monitor lizards.
**Terrestrial**	Potential terrestrial threat, including mammalian land predators but also running antelopes and warthogs. Confirmed predators present at the site include leopards and caracals (seen on camera traps). Potential but unlikely predators include jackals and poaching dogs (observed while following monkeys). Note that predators are notoriously hard to confirm, meaning that if researchers could not identify any clear stimulus responsible for the calls, such events had to be classified as unknown (see below).
**New male**	Sighting of an unknown adult male in the group.
**Unknown**	All barking events in which context could not be clearly determined. May include all the above categories and potential displays of males. Although male behaviour occasionally appeared to suggest a display, it could never be excluded that human observers had missed a predator or aggressive context. We therefore did not include 'male display' as a category since it could never have been scored without uncertainty. Potential displays may involve shaking branches, jumping into a tree and barking from an elevated position while other monkeys seemed unconcerned. However, such behaviour also occurred in confirmed predator cases and aggressive interactions.

Table S8 Definitions for age categories.

Age category	Definition
**Adult female**	Females were considered adults as soon as they were four years old, which is the typical age of first reproduction.
**Adult male**	Males were considered adults as soon as they dispersed for the first time, which typically happens when they are four to five years old.
